# Electrospinning of Natural Polymeric Fibers with Essential Oils for the Control of Multidrug-Resistant Pathogens

**DOI:** 10.3390/polym18080972

**Published:** 2026-04-16

**Authors:** Deysi Alejandrina Cabrera Segura, Verónica Santacruz Vázquez, Sandra Mendoza, Santa Toxqui-López, Paulina Arellanes-Lozada, Claudia Santacruz Vázquez

**Affiliations:** 1 Facultad de Ingeniería Química, Benemérita Universidad Autónoma de Puebla, Av. San Claudio y 18 Sur, Puebla 72570, Mexico; cs224570056@alm.buap.mx (D.A.C.S.); veronica.santacruz@correo.buap.mx (V.S.V.); santa.toxqui@correo.buap.mx (S.T.-L.); 2 Facultad de Química, Universidad Autónoma de Querétaro, Cerro de Las Campanas s/n, Querétaro 76010, Mexico; smendoza@uaq.mx; 3 Gerencia de Investigación en Explotación, Instituto Mexicano Del Petróleo, Eje Central Lazaro Cardenas 152, San Bartolo Atepehuacan, Gustavo a. Madero, Mexico City 07730, Mexico

**Keywords:** essential oils, electrospun fibers, multidrug-resistant, polymeric fibers, antimicrobial activity

## Abstract

Antimicrobial resistance (AMR) represents one of the major threats to global health, driven by the indiscriminate use of antibiotics and decline in the development of new therapeutic agents. In this context, essential oils (EOs) have emerged as innovative natural alternatives due to their broad-spectrum antimicrobial activity and low potential to induce bacterial resistance. However, their clinical application is limited by their volatility, low chemical stability, and rapid degradation. The incorporation of EOs into electrospun natural polymer fibers has emerged as an effective strategy to overcome these limitations, improving their stability, enabling controlled release, and enhancing their antimicrobial efficiency. This review focuses on the use of electrospun natural polymers for biomedical applications, highlighting their biocompatibility, biodegradability, and ability to mimic the extracellular matrix, thereby promoting cell interaction. Additionally, their high surface area and porous structure facilitate efficient encapsulation and controlled release of bioactive compounds. Recent advances in the development of these systems against clinically relevant multidrug-resistant pathogens are analyzed, along with the antimicrobial mechanisms of EOs. Finally, the factors influencing encapsulation and release efficiency, as well as the main challenges and future perspectives for clinical translation, are discussed.

## 1. Introduction

**Figure 1:**
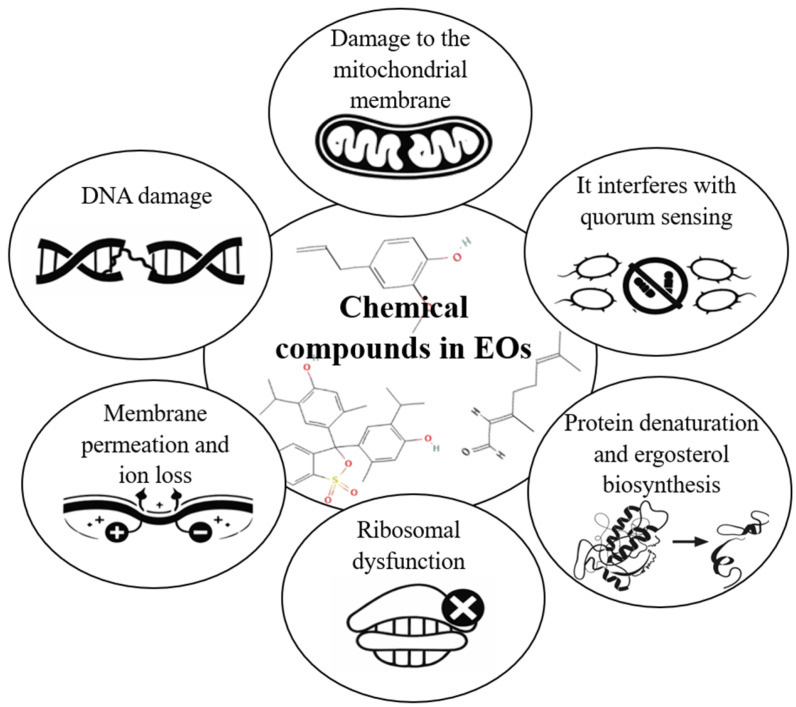
Action mechanisms of EOs against bacterial cellular components [14].

Antimicrobial resistance (AMR) is a growing global health crisis, with projections estimating up to 10 million deaths annually by 2050 if new therapeutic strategies are not developed [1,2,3,4]. This phenomenon is primarily attributed to the indiscriminate use of antibiotics, limited innovation in the development of new drugs, and environmental dissemination of resistance genes [1,5]. Among priority pathogens, that have developed multidrug-resistant (MDR) are *Pseudomonas aeruginosa* (*P. aeruginosa*), *Acinetobacter baumannii* (*A. baumannii*), *Klebsiella pneumoniae* (*K. pneumoniae*), *Escherichia coli* (*E. coli*), *Staphylococcus aureus* (*S. aureus*), *Enterococcus faecium* (*E. faecium*), and *Enterobacter* spp., which exhibit complex resistance mechanisms, including the action of efflux pump, production of inactivating enzymes, alteration of therapeutic targets, and a remarkable ability to form biofilms, persist in hospital environments, and transfer resistance genes [4,5,6,7,8,9]. Previously, these bacteria were mainly associated with nosocomial infections; however, they have now spread into community settings. If this trend continues, large-scale untreatable infections may emerge, posing critical challenges to healthcare systems. Therefore, the development of new therapeutic approaches to contain and mitigate the dissemination of AMR is urgently needed [4,9]. In this context, essential oils (EOs) have recently gained increasing attention as alternative antimicrobial agents due to their multiple mechanisms of action, which reduce the likelihood of resistance development [10]. EOs are oily, aromatic compounds produced by plants as secondary metabolites. Their chemical composition mainly consists of mixtures of terpenes, phenols, and aldehydes, which are responsible for their antimicrobial activity through multiple mechanisms, including disruption of cell membranes, inhibition of enzymes, metabolic interference, coagulation of cytoplasmic contents, and alteration bacterial quorum sensing (see Figure 1) [11,12,13,14]. Several studies have reported that EOs derived from oregano (*Origanum vulgare*, *O. graveolens*) (OEO), thyme (*Thymus* spp.) (TEO), clove (*Syzygium aromaticum*) (CLEO), tea tree (*Melaleuca alternifolia*) (TTEO), cinnamon (*Cinnamomum verum*) (CEO), lemongrass (*Cymbopogon citratus*) (LEO), and mint (*Mentha* spp.) (MEO) exhibit antimicrobial activity against MDR organisms [15]. Notwithstanding, the direct application of EOs is limited because of their high volatility, low aqueous solubility, and susceptibility to oxidative degradation. In this regard, the development of controlled release systems—particularly through electrospinning techniques has emerged as an effective strategy to enhance their stability, bioavailability, and therapeutic efficacy [16,17,18].

Electrospinning enables the production of polymeric fibers with high surface-to-volume ratio and the ability to encapsulate bioactive compounds. Nevertheless, despite advances in its application, the use of natural polymers (such as chitosan, alginate, gelatin, and cellulose) in electrospun fibers loaded with EOs still faces important challenges [19,20,21,22]. Among the main knowledge gaps, the exploration of electrospun fibers loaded with a limited variety of EOs stands out, as most studies have been focused on a relatively small number of compounds. This fact restricts the understanding of the antimicrobial activity of different active compounds in EOs within natural polymeric matrices [23,24,25]. Furthermore, there are few studies evaluating these systems against MDR clinical strains, while various studies analyze laboratory strains under controlled conditions, limiting their translational relevance [26,27,28,29,30,31,32,33,34,35,36]. Taken together, these limitations highlight the need for more comprehensive studies that include a broader diversity of EOs, polymeric matrices, and clinically relevant microbiological models.

Electrospun formulations incorporating EOs represent an inherent challenge due to the complex interdependence of physicochemical and processing factors. These systems are governed by weak non-covalent interactions, such as hydrogen bonding and Van der Waals forces, which limit the ability to achieve structural control over the system [37]. In addition, the difference in polarity between hydrophobic EOs and hydrophilic polymeric matrices promotes incompatibility and phase separation, leading to heterogeneous structures that compromise encapsulation efficiency, release profiles, and reproducibility [38,39]. Moreover, process reproducibility is also affected by solution parameters, operational conditions, and environmental factors that affect fiber diameter, morphology, and distribution of the active compound [40,41,42,43]. These structural and physicochemical characteristics directly impact antimicrobial activity, since non-uniform distribution and uncontrolled release of EOs may result in sub-inhibitory concentrations or premature release, thereby reducing their effectiveness [44,45,46]. Given the above, the standardization of formulations and processes poses a significant barrier to the development of EO-loaded electrospun fibers on an industrial scale and their eventual clinical application [22,37,47].

The evidence regarding the antimicrobial properties of electrospun fibers loaded with EOs at the preclinical and clinical levels is currently limited. While various studies, both in vitro and in vivo, have demonstrated the potential of EOs and electrospun natural polymeric fibers loaded with EOs, existing research has focused primarily on in vitro assays. This preponderance of laboratory studies is in contrast with the scarcity of work evaluating their safety, toxicity, and efficacy in relevant animal models or in controlled clinical trials [23,29,33,46,48,49,50,51]. As a result, it is not possible to establish solid correlations between experimental results and actual therapeutic performance in humans [33,34,51]. This translational gap represents a critical barrier to their biomedical implementation.

The aim of this review is to analyze the state of the art in the development of electrospun natural polymeric fibers loaded with EOs that have exhibited antimicrobial activity against MDR pathogens. Given the inherent limitations of EOs, their incorporation into electrospun natural polymer fibers offers a promising strategy to improve their stability, enable controlled release, and enhance antimicrobial activity. In this context, this review also examines the key factors influencing encapsulation efficiency and release kinetics, as well as the antimicrobial mechanisms of EOs against clinically relevant MDR strains. Finally, the current challenges and future perspectives for the clinical translation of these bioactive systems are discussed.

## 2. Electrospinning Technology for Fibers Used in the Biomedical Field

**Figure 2:**
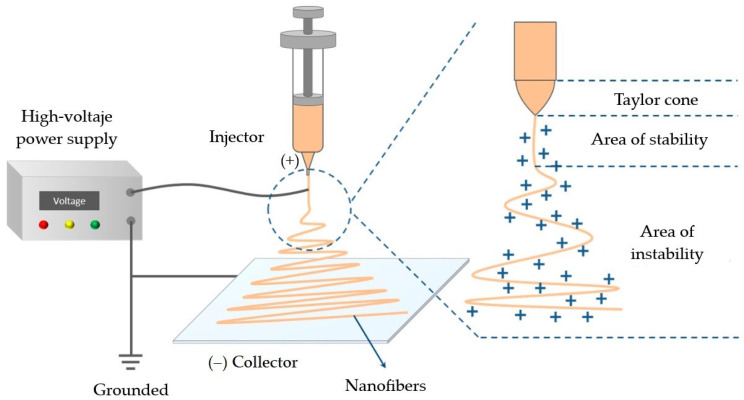
Schematic representation of an electrospinning system [41].

Electrospinning has generated significant interest due to its versatility as a technique for producing continuous micro and nanofibers from polymer solutions [19,20]. The fibers obtained through this technique are characterized by their high surface to volume ratio, high porosity, and remarkable ability to encapsulate bioactive compounds [21,22]. These properties facilitate the protection of sensitive molecules and enable the controlled release of therapeutic agents, making electrospinning a particularly attractive strategy for the development of advanced systems with biomedical applications [7,13,52,53]. An electrospinning system consists of three components: a nozzle containing the polymer solution, a collection plate, and a power source [54]. This method employs a high-voltage electric field between the injector and collector. The polymer solution contained in the injector generates a droplet at its outlet that is affected by the electromagnetic field, inducing the formation of a fine jet which stretches and solidifies after the solvent evaporates, resulting in fibers (see Figure 2) [18,26,40,55].

The final properties of electrospun fibers are primarily determined by the polymer solution composition, operating parameters, and environmental conditions during the electrospinning process (see Table 1) [40,41,42].

Among these factors, the concentration and molecular weight of the polymer play a decisive role, as they directly influence the solution viscosity and jet stability during the process [57,58,59]. It has been reported that increasing the polymer concentration and molecular weight leads to an increase in solution viscosity due to enhanced polymer chain entanglement. This, in turn, promotes the formation of a more stable and continuous jet during electrospinning, resulting in fibers with more homogeneous, continuous morphologies. Additionally, higher viscosity is associated with an increase in both average fiber diameter and pore size, along with a reduction in structural defects such as beads and irregularities, which are commonly observed in low-concentration and low-molecular-weight solutions characterized by low viscosity and surface tension [44,60,61,62,63]. Therefore, in the electrospinning process, each polymer has an optimal viscosity range, which largely determines the formation of continuous fibers and their final morphology [59].

**Figure 3:**
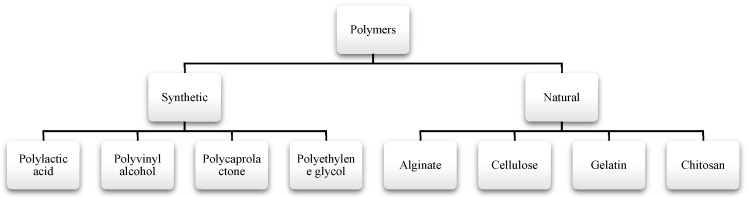
Polymers commonly used in electrospinning processes [18,44,64,65,66,67,68].

In this context, the selection of polymers for the electrospinning process is critical, and the materials used are generally classified as synthetic or natural, as shown in Figure 3 [64,65,66,67]. Synthetic polymers possess a uniform chemical composition, high electrical conductivity, high mechanical stability, and good processability. Nonetheless, their limited biocompatibility and slow degradability remain significant technological challenges [64,65,66,67,68]. Although natural polymers offer enhanced biocompatibility, lower immunogenicity, and inherent biodegradability, they typically present lower mechanical strength and reduced structural stability [44,64,65,66,67,68]. The combination of both types of polymers enables the development of materials with optimized properties for biomedical applications, for instance, by promoting improved cell adhesion and proliferation when used in tissue engineering [26,44,64,65,66,67,68].

Electrospun fibers have gained significant relevance in the biomedical field due to their high porosity, biocompatibility, biodegradability, and ease of functionalization. These characteristics allow them to mimic the three-dimensional architecture of the extracellular matrix and promote tissue regeneration [69,70,71,72]. The ability to encapsulate active compounds or functionalize their surface is among the most notable properties of electrospun polymeric fibers, which enhances the interactions with microorganisms due to their nanoscale dimensions, and that are comparable to those of many biological molecules [72,73,74]. This nanoscale feature facilitates the simulation of natural biological environments and improves key properties such as stability, multifunctionality, bioavailability, controlled release, and adhesion of bioactive compounds [40,75,76]. Furthermore, their highly porous structure allows the incorporation of various substances, including drugs, specific molecules, or bioactive compounds, which can be released in a sustained and controlled manner. This enhances their diffusion and interaction with pathogenic microorganisms, thereby prolonging their therapeutic effect and reducing adverse effects [77,78,79].

These characteristics have enabled their application as scaffolds for tissue engineering and bone regeneration, where modifications through the incorporation of hydroxyapatite, polydopamine, or biocompatible metallic coatings enhance hydrophilicity, mechanical properties, and cellular activity [80,81,82]. Their high surface area allows the integration of antimicrobial agents such as nanoparticles, anti-inflammatory agents, and natural antioxidants, including curcumin, green tea extract, resveratrol, vitamin C, vitamin E, flavonoids, and glutathione. These agents help eliminate free radicals, reduce inflammation, and accelerate healing processes [61,83,84]. Moreover, electrospun fibers possess adhesive properties that enable them to adhere effectively to the wound surface for extended periods, acting as a protective barrier against the external environment and reducing the risk of infection, making them highly promising materials for the development of advanced wound-healing dressings [85,86,87,88]. Taken together, these properties have shown great potential in the treatment of complex wounds, including burns and diabetic ulcers, where they help control infections, promote angiogenesis, and improve skin tissue regeneration [89,90]. These characteristics make electrospun fibers highly promising platforms for incorporation of EOs with the aim of developing materials with antimicrobial activity for biomedical applications [18,91,92].

## 3. EOs as Antimicrobial Agents

**Figure 4:**
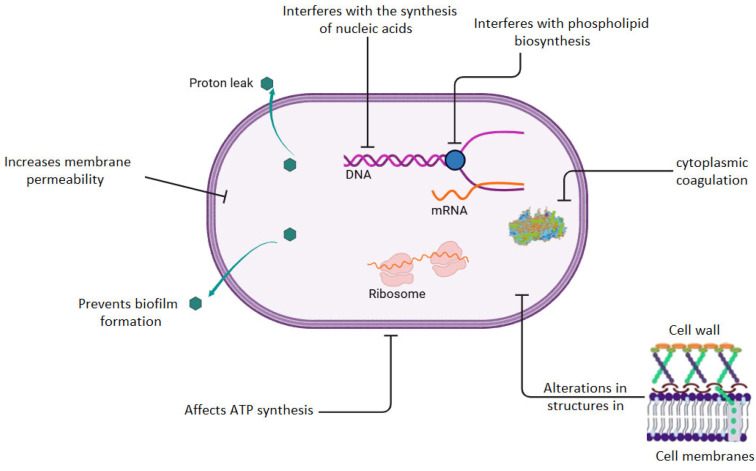
Schematic representation of structural and functional alterations induced by EOs in bacterial cells.

EOs are complex mixtures of volatile organic compounds, generally aromatic in nature, primarily extracted from different parts of plants, including seeds, leaves, stems, flowers, shoots and bark [9,15,93]. Their recognized antibacterial activity is associated with the high concentration of bioactive molecules in their composition, particularly phenols, monoterpenes, sesquiterpenes, and various terpenoids, which are responsible for their biological properties [94]. Certain phenolic compounds present in EOs contain hydroxyl groups (-OH) capable of forming hydrogen bonds with enzymatic active sites, such as methylesterases and synthases, promoting the generation and accumulation of reactive oxygen species (ROS) [95,96]. This process induces oxidative stress, leading to bacterial cell death through growth inhibition, disruption of membrane integrity, and DNA damage, as shown in Figure 4 [59,96]. Additionally, the lipophilic nature of EOs facilitates their diffusion through the phospholipid bilayer of microorganisms. This process induces alterations in cell membrane permeability through interaction with structural and transport proteins, resulting in the leakage of intracellular components and inhibition virulence factors associated with quorum sensing [96,97,98,99,100].

Other mechanisms described for bioactive compounds in EOs include the reduction in intracellular pH and disruption of the electrochemical potential, affecting ATP and nucleic acid synthesis. Furthermore, these compounds can compromise cell membrane integrity and interfere with the biosynthesis of lipoteichoic acid, a structural component of the cell wall in Gram-positive bacteria, leading to inhibition of bacterial growth or cell death (see Figure 4) [101,102]. In addition, studies have shown that the active compounds in EOs can exert a synergistic effect, making them more effective against pathogens in the ESKAPE group (*E. faecium*, *S. aureus*, *K. pneumoniae*, *A. baumannii*, *P. aeruginosa*, and *Enterobacter* spp.) [98,103,104,105,106,107,108,109,110,111,112,113,114,115,116,117,118,119,120,121,122,123,124,125,126,127,128,129,130,131,132,133,134,135,136,137,138,139,140,141,142,143,144,145,146,147,148,149,150,151,152,153,154,155,156,157,158,159].

In this context, various EOs, including those derived from TEO, OEO, CLEO, CEO, TTEO, LEO, and MEO, have demonstrated high activity against multidrug-resistant microorganisms, including strains producing extended-spectrum β-lactamases, carbapenemases, and clinical isolates with high biofilm-forming capacity [98,105,107,108,109,110,111,112,113,114,115,116,117,118,119,120,121,122,123,124,125,126,127,128,129,130,131,132,133,134,135,136,137,138,139,140,141,142,143,144,145,146,147,148,149,150,151,152,153,154,155,156,157,158,159]. The following sections will individually address the available scientific evidence for each one of these EOs, analyzing their chemical composition, action mechanisms, and performance against clinically relevant pathogens [107].

LEO, obtained primarily from *Cymbopogon citratus* and *Cymbopogon flexuosus*, is distinguished by a unique phytochemical profile dominated by citral isomers, which account for 75–85% of its composition. The aldehyde functional group of citral is highly reactive with membrane lipids and proteins, inducing structural modifications. Other components, such as geraniol, improve membrane permeability and fluidity, facilitating the leakage of cytoplasmic contents, while linalool and citronellal act synergistically to potentiate the activity of citral [107,108,109,110,111]. This composition confers broad-spectrum antimicrobial activity, with particularly pronounced and consistent effects against *P. aeruginosa*, *S. aureus*, *E. faecalis*, *L. monocytogenes*, *S. paratyphi*, *E. aerogenes*, and *P. vulgaris* [111,112]. The dominant mechanism of LEO in Gram-negative bacteria involves its ability to penetrate and destabilize cytoplasmic membranes, causing the leakage of ions and metabolites, impairing ATP production, and inducing cytoplasmic coagulation, ultimately leading to cell death [87]. In Gram-positive bacteria, it has also been observed to disrupt the peptidoglycan layer, compromising cell wall integrity and promoting the uncontrolled release of intracellular components [107,113]. Furthermore, the high citral content, together with other monoterpenes present in LEO, can interfere with the adhesion and maturation processes of biofilm formed by *S. aureus*, *E. coli*, *P. aeruginosa*, and *L. monocytogenes* [114,115]. It has been studied that LEO has high content of citral, β-myrcene, limonene, geranyl acetate, and linalool, and it has demonstrated antimicrobial activity against *Staphylococcus* spp. and *E. coli*, primarily related to the inhibition of the electron transport chain and blockade of ATP synthesis [116].

Similarly, MEO, primarily obtained from *Mentha* spp., contains oxygenated compounds with C-2 (carvone) or C-3 (menthone, piperitone, piperitene, and pulegone) functional groups, characteristic of oxygenated monoterpenes bearing free hydroxyl groups. These compounds promote the formation of delocalized electrons, acting as proton exchangers that reduce the pH gradient and destabilize the cytoplasmic membrane. This leads to disruption of the proton motive force, subsequent ATP depletion, and ultimately cell death [117]. These mechanisms explain its broad antimicrobial activity against *E. coli*, *S. aureus*, *K. pneumoniae*, *P. mirabilis*, *P. aeruginosa*, *A. baumannii*, *S. pyogene*, and *C. albicans* [118,119]. The most potent antibacterial constituent of MEO has been identified as menthol, which increases membrane permeability, causing leakage of cytoplasmic contents and leading to bacterial cell death [120]. Additionally, menthone has demonstrated activity against methicillin-resistant *S. aureus* (MRSA) by altering the depolarizing potential and the integrity of the cell membrane [116,119]. It is worth noting that other compounds in MEO, such as β-pinene, limonene and menthone, have displayed activity against clinically relevant pathogens, including *A. baumannii*, *P. aeruginosa*, *S. aureus*, *L. monocytogenes*, *C. albicans*, and *A. niger.* These compounds have been found to inhibit virulence factors such as toxins, elastase, proteases, pyocyanin, and chitinase [105].

TEO, primarily obtained from *Thymus vulgaris*, is characterized by the presence of oxygenated monoterpenes, with carvacrol as the predominant compound, followed by thymol and borneol, along with lower amounts of linalool, α-terpineol, 1,8-cineole, and p-cymene [121,122,123]. Some studies suggest that thyme-derived compounds interact with cell membranes through hydrogen bonding, disrupting the integrity of the lipid bilayer, increasing permeability, and causing the leakage of intracellular contents and ions [124]. Carvacrol has been shown to cause the release of lipopolysaccharides from the membrane, while thymol binds to membrane proteins, interfering with their synthesis and altering membrane permeability, thereby contributing to microbial death [125]. In addition, the efficacy of TEO against Gram-positive and Gram-negative bacteria has been demonstrated, including *E. coli*, *Enterobacter* spp., *E. faecalis*, *P. aeruginosa*, *Klebsiella* spp., and *Staphylococcus* spp. Its antimicrobial activity is associated with the inhibition of enterotoxin production, alterations in membrane fatty acid composition, and interference with quorum sensing systems and biofilm-forming organisms [126].

As mentioned above, TEO, LEO and MEO have been shown to exhibit antimicrobial activity against *Staphylococcus* spp. and *E. coli* [127]. This activity is associated with multiple mechanisms of action, including the disruption of cytoplasmic membrane integrity, inhibition of ATP synthesis, interference with the electron transport chain, and reduction in the production of virulent factors such as toxins, elastase, proteases, pyocyanin, and chitinase [105,116,126]. Moreover, the antimicrobial activity of MEO (*Mentha piperita* L.) against methicillin-resistant *S. aureus* (MRSA) has been evaluated, revealing a potent inhibitory effect ascribed to disruption of bacterial membrane potential and a decrease in exotoxin production [126,128]. These findings suggest that EOs can interfere with key physiological processes involved in bacterial virulence. Additionally, TEO and CLEO have been reported to exhibit broad spectrum activity against carbapenem-resistant *P. aeruginosa*, *A. baumannii*, and *K. pneumoniae* [129].

Another EO, such as CLEO obtained from *Syzygium aromaticum*, is known for its potent antimicrobial activity, which is attributed to its main components: eugenol, caryophyllene, and 2-(octadecyloxy)ethanol. However, it also contains other active components at lower concentrations, including pinene (α and β), neral, geranial, γ-terpinene, cis-ocimene, allo-ocimene, 1,8-cineole, linalool, borneol, myrcene, and pinene-2-ol [130,131]. The hydrophobic nature of CLEO enables it to interact with the cytoplasmic membrane of *S. aureus* (MRSA), compromising its structural and functional integrity, which leads to leakage of intracellular components and disruption of cellular metabolism [130,132,133]. In this context, eugenol, the main bioactive component of CLEO, has been shown to decrease the expression of the regulatory gene *sarA*, the enterotoxin-encoding gene *seA*, and the adhesion gene *icaD*. These genes play a fundamental role in bacterial colonization and biofilm formation [134,135,136].

In contrast, the main compounds found in CEO are cis-cinnamaldehyde, eugenol, cinnamyl acetate, linalool, β-caryophyllene, benzyl benzoate, eugenol acetate, coumarin, and inorganic elements. These compounds have exerted antibacterial effects against *S. aureus*, *Salmonella*, *E. coli*, and *P. aeruginosa* [137,138]. The action of CEO on the bacterial cell wall alters its protective structure and can lead to bacterial cell death, a process associated with the compound cis-cinnamaldehyde. Furthermore, high concentrations of CEO have been shown to cause significant morphological changes, such as swelling and rupture of bacterial cells, leading to the release of intracellular potassium ions (K^+^), indicating membrane damage. The lipophilicity of the EO facilitates its penetration into the bacterial membrane, altering its properties and improving its permeability [137,139]. Such high concentrations also increase ROS production, causing severe oxidative damage in *S. Enteritidis* strains [140,141,142,143]. In addition, the effect of CEO on the gene expression of outer membrane proteins in *S. Enteritidis* has been studied. This type of proteins plays a vital role in bacterial metabolism, regulation of drug uptake, maintenance of cell shape, and synthesis of related metabolites. These studies show that CEO increases the permeability of these proteins, disrupts bacterial metabolism, and highlights its potential as a natural antibiotic alternative [137,143].

Additionally, studies based on nanoemulsions of CLEO and CEO have demonstrated antibiofilm activity and a significant reduction in the viable cell count of *S. aureus* with intermediate susceptibility to vancomycin [144,145]. Similarly, both EOs have shown broad-spectrum activity against various clinically relevant pathogens and their biofilms, including *S. aureus*, *S. epidermidis*, *E. faecalis*, *S. pyogenes*, *E. coli*, *P. aeruginosa*, *P. mirabilis*, *K. pneumoniae*, and *C. albicans* [146]. The antimicrobial mechanisms associated with these EOs are multifactorial and include the denaturation of structural and enzymatic proteins, loss of intracellular metabolites, cytoplasmic coagulation, inhibition of motility, and interference with bacterial communication systems (quorum sensing), which contributes to the inhibition of biofilm formation and maintenance [17,90].

Furthermore, OEO (*Lippia graveolens* or *Origanum vulgare*) has demonstrated broad-spectrum antimicrobial activity at low concentrations against clinically relevant pathogens, including *Staphylococcus* spp., *S. aureus*, *Klebsiella* spp., *E. faecalis*, *A. baumannii*, and *S. typhi* [123,146]. The chemical composition of this substance is the primary factor contributing to its unique properties. It contains a variety of chemical compounds, including thymol, p-cymene, carvacrol, β-caryophyllene, 1,8-cineole, and γ-terpinene, as referenced in different studies [9,147,148]. These metabolites exert their antimicrobial effect through multiple mechanisms of action, including the inhibition of essential bacterial enzymes and reduction in the activity of virulence factors, such as coagulase and bacterial lipase, thereby contributing to a decrease in microbial infectivity potential [9,147]. One of the compounds that is inserted into the lipid bilayer is carvacrol, which causes an increase in cell membrane permeability, leading to the leakage of ions and vital cellular contents. Furthermore, this phenolic compound has been shown to act as a proton exchanger, disrupting the proton motive force and ATP synthesis in bacteria [98,149]. It has been reported that OEO and TEO have inhibited quorum sensing pathways and biofilm formation [149,150,151].

Finally, TTEO is composed primarily of terpinen-4-ol, 1,8-cineole, and terpinolene [152]. Terpinen-4-ol has been identified as the most effective compound against multidrug-resistant microorganisms, including glucopeptide-resistant enterococci, aminoglycoside-resistant *Klebsiella* spp., and *P. aeruginosa*. It also exerts antimicrobial effects against *Staphylococcus* spp., *Streptococcus* spp., *E. coli*, *K. pneumoniae*, *E. faecalis*, and *C. albicans* [152,153,154]. Moreover, TTEO has demonstrated efficacy against various bacterial and fungal strains, including *E. coli*, *S. aureus*, *C. albicans*, and *Aspergillus niger*, by penetrating their cell walls and cytoplasmic membranes, resulting in the loss of cytoplasmic content and cell death [152,155,156]. It has also inhibited bacterial cell division, contributing to its efficacy against microorganisms such as *Staphylococcus* spp. and *E. coli* [90]. In addition, TTEO has suppressed the expression of the *Agr* gene by inhibiting the *AgrA* regulator, leading to the inhibition of biofilm formation in *S. aureus* (MRSA). In addition, this EO set itself as a promising adjuvant agent, enhancing the efficacy of treatment for drug-resistant infections by potentiating antimicrobial activity against multidrug-resistant bacterial pathogens [157,158]. A comparative study evaluated the antimicrobial activity of TTEO, TEO, CLEO, and CEO against five clinically relevant pathogens: *P. aeruginosa*, *MRSA*, *S. pneumoniae*, *K. pneumoniae*, and *E. coli*. The results demonstrated that TTEO and TEO exhibited the highest efficacy in cell membrane degradation assays. In addition, both oils exhibited a synergistic effect when used in combination with antibiotics such as gentamicin and vancomycin, reducing the effective concentration of these drugs against *P. aeruginosa* and MRSA [159].

Overall, the analyzed studies highlight the potential of EOs as natural antimicrobial agents for the development of targeted strategies to control multidrug-resistant pathogens associated with nosocomial infections. However, further research is still required to better understand their mechanisms of action, optimize their delivery systems, and evaluate their feasibility for clinical application [160,161]. In this context, the objective is to enhance the stability, bioavailability, and controlled release of EOs, including TEO, OEO, CLEO, CEO, TTEO, LEO and MEO, which have exhibited significant antimicrobial activity against MDR pathogens, including bacteria belonging to the ESKAPE group [160,162,163,164].

## 4. Incorporation of Eos into Electrospun Fibers

**Figure 5:**
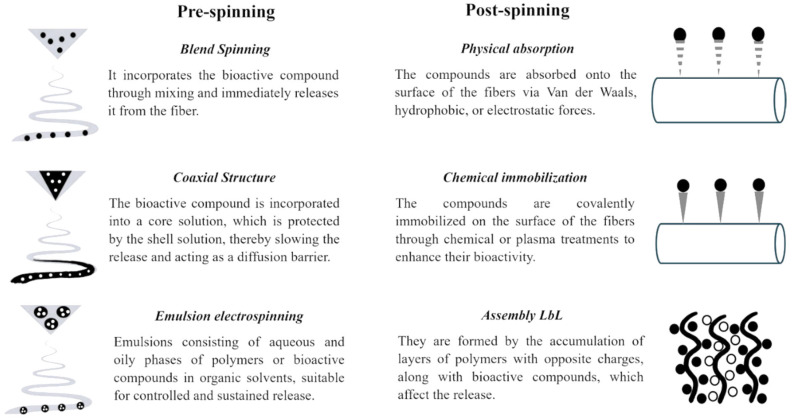
Pre-spinning and post-spinning strategies for incorporating bioactive compounds into electrospun fibers [17,167,168,169].

The incorporation of EOs into polymeric matrices offers significant advantages, including improved chemical and thermal stability, reduced volatility, enhanced dispersion and solubility in water, and minimized degradation due to environmental factors such as oxygen and light [17,165,166]. Collectively, these effects enhance the stability and bioavailability of the active compounds, thereby boosting the antimicrobial efficacy of EOs in electrospun fiber-based systems [18,59,60]. This integration can be achieved through pre-spinning or post-spinning strategies, as demonstrated in Figure 5 [21,167,168,169]. Notwithstanding, the addition of EOs has been shown to alter the physicochemical properties of the polymer solution, particularly viscosity, conductivity, and surface tension. These parameters directly influence the fiber formation process [27,28]. As a result, variations in the final morphology may occur, such as increases in fiber diameter, bead formation, increased surface roughness, or the generation of pores. These structural modifications influence the mechanical and functional properties of the fibers, affecting the encapsulation efficiency and the release kinetics of the bioactive agent [23,57].

## 5. Morphology and Physicochemical Properties of Electrospun EO Fibers

The morphology of electrospun fibers is determined by a complex set of operational and physicochemical parameters. Key factors include voltage, solution viscosity, surface tension, and electrical conductivity, as well as the distance between the needle and collector (see Table 2). The incorporation of EOs at different concentrations can modify one or more of these parameters, thereby altering the fiber morphology [43,44,56]. The final morphology is the result of a delicate balance between viscoelectric and tensile electrostatic forces. Consequently, any variation in the polymer solution can directly affect the repulsive forces responsible for jet elongation [44,170,171]. In this context, both a decrease and an increase in fiber diameter have been reported following the addition of EOs [27,28]. For example, Unalan et al. (2019) [29] demonstrated that an increase in EO concentration can induce progressive growth in fiber diameter. This effect is generally associated with an increase in the solution viscosity. However, in other systems, a reduction in fiber diameter has been observed. This behavior has been attributed to changes in the electrical conductivity and/or surface tension of the polymer solution [30,31,32,33]. Table 2 summarizes the impact of the composition of polymer solutions on electrospun fibers. It considers different polymer and EO concentrations, as well as their direct effect on the morphology and diameter of the fibers.

Some studies presented in Table 2 indicate that the addition of EOs can lead to an increase in fiber diameter; notwithstanding, this effect largely depends on the polymeric system and processing conditions. In a study, a polymeric solution was formulated using MEO (*Mentha longifolia* L.) at 0.5%, 1%, and 2% in electrospun carboxymethylcellulose-gelatin (CMC–GE). This solution was then processed via electrospinning under an applied voltage of 25 kV, a flow rate of 0.5 mL/h, and ambient temperature. As a result, smooth, fine, and uniform fibers with bead shape were produced. These morphological characteristics were attributed to lower solution viscosity, along with increased surface tension and electrical conductivity compared to the control group (CMC-GE) [28]. The average diameters of fibers loaded with 0.5%, 1%, and 2% of MEO were measured at 393.56, 414.29, and 454.76 nm, respectively, showing a clear increase in diameter with growing MEO concentration. This trend suggests that the incorporation of MEO affects polymer chain entanglement and interactions within the CMC–GE matrix, thus contributing to the formation of thicker fibers [28]. Additionally, the combined effect of high surface tension and elevated electrical conductivity significantly affected jet stability during the electrospinning process. This promoted a transition from smooth, homogeneous nanofibers to bead-on-string structures [28,29].

Hameed et al. (2021) [34] demonstrated that electrospun fibers were composed of chitosan (CS) and polyethylene oxide (PEO) at 5% in a 1:1 ratio, loaded with CEO at 0.5% and 1%. These fibers exhibited average diameters of 154 and 189 nm, respectively. This behavior was attributed to a reduction in electrical conductivity and an increase in solution viscosity associated with higher CS content [34]. Notably, only a 50:50 CS/PEO ratio yielded smooth, bead-free fibers. As the CS concentration increased, the solution became progressively more viscous and difficult to electrospin, resulting in the formation of bead-like structures [29].

In another study, electrospun fibers based on 10% (*w*/*w*) hydrolyzed collagen loaded with 10% CLEO or CEO exhibited well-defined fibers with porous three-dimensional structures [172]. The results demonstrated that the average diameter varied depending on the support: leather (404.8–463.2 nm) > cotton (485.2–683.1 nm) > waxed paper (531.2–717.6 nm). This demonstrates that the selection of substrate for the deposition of electrospun fibers is of paramount importance. The type of EO used also had a significant impact on the diameter of the nanofibres. On leather, the average diameters were found to be 531.2, 568.3, and 717.6 nm for collagen hydrolysate without EO, loaded with CLEO, and loaded with CEO, respectively [172]. Ansarifar and Moradinezhad (2022) [35] developed electrospun zein fibers at 30% (*w*/*v*) with TEO at 4% (*v*/*v*). The scanning electron microscopy (SEM) images of zein fibers with and without TEO exhibited uniform, homogeneous, bead-free, and smooth surface morphologies. Meanwhile, the fiber diameters were 195.0 ± 32.1 nm and 402.3 ± 26.6 nm in the absence and presence of TEO, respectively. This increase in fiber diameter was attributed to the reduction in electrical conductivity caused by EO addition, which decreases polymer jet elongation under the applied electric field and leads to thicker fibers.

In a study on electrospun fibers composed of polyvinyl alcohol (10%) and gellan (1%), an average fiber diameter of 204.03 nm was reported. Following the incorporation of cinnamaldehyde (a phytoactive molecule present in CEO) into the polymer solution, increase in viscosity was observed; resulting in fibers with larger diameter (278.5 nm). Additionally, a non-uniform distribution of cinnamaldehyde within the fibers was identified, which could be attributed to its hydrophobic nature [106]. The addition of cinnamaldehyde led to a decrease in the electrical conductivity and an increase in the solution viscosity, which promoted a higher degree of polymer chain entanglement. In electrospinning systems, a solution with higher viscosity and greater chain entanglement, together with lower conductivity, requires a stronger electrostatic driving force to be stretched. This limits jet thinning and ultimately results in the formation of fibers with larger diameters [106].

In contrast, some studies have reported a reduction in fiber diameter upon the addition of EOs. For instance, Amarante et al. (2023) [30] examined electrospun cellulose acetate (CA) fibers with PEO with and without the incorporation of CEO, CLEO and EEO. Their findings indicate the absence of microspheres, attributable to adequate solvent evaporation, resulting in regular cylindrical structures. The measured fiber diameters were 2.42, 2.13, 1.98, and 1.90 µm for neat CA and CA loaded with CEO, CLEO, and EEO, respectively. These results demonstrate a slight reduction in fiber diameter upon EO incorporation, suggesting that the presence of EOs can modulate fiber morphology, likely through alterations in solution properties such as viscosity and surface tension. In another study, PEO nanofibers were electrospun using different LGEO concentrations, identifying optimal processing conditions at 20 kV and a tip-to-collector distance of 18 cm. The results showed uniform, defect-free fibers with average diameters of 0.81 μm for neat PEO; and 0.61, 0.57, and 0.54 μm for fibers containing 3%, 6%, and 9% LGEO, respectively. A progressive reduction in fiber diameter was observed with increasing LGEO concentration, attributed to a decrease in apparent viscosity and an increase in the electrical conductivity of the emulsion. These changes enhanced jet stretching during electrospinning, leading to the formation of thinner fibers [32]. De Souza et al. (2024) [33] evaluated a blend of Ecovio^®^ (EC) polymers, composed of PLA (55%) and PBAT (45%), incorporating 0.5, 1, and 1.5 mL of TTE. The fibers without TTE exhibited beads and heterogeneous structures with average diameter of 478 nm. The addition of TTE reduced surface tension and viscosity and facilitated the elongation of the polymer mixture jet. This led to thinner and more uniform fibers with diameters of 393, 252, and 278 nm corresponding to 0.5, 1, and 1.5 mL of TTE, respectively. The slight increase in the average fiber diameter of 252 to 278 nm could be attributed to the higher surface tension; however, the difference was not statistically significant. Similar results were obtained by electrospinning 14% (*w*/*v*) PLA, producing fibers with average diameters of 1002 nm, and reducing the diameter to 633 nm by loading 10% (*v*/*v*) limonene EO [31]. Studies reporting a reduction in fiber diameter after EO incorporation typically share similar electrospinning parameters, including applied voltages of 10–12 kV, flow rates of 0.5–0.7 mL/h, and tip-to-collector distances of 10–14 cm. Notably, these experiments were consistently conducted under ambient conditions, with temperatures not exceeding 25 °C [30,31,33].

In a comprehensive study, Gilan et al. (2025) [37] evaluated the influence of TEO concentration, tip-to-collector distance, and applied voltage on the morphology of thermoplastic polyurethane (10%) nanofibers. The incorporation of 5% and 10% TEO led to bead formation, attributed to the low solution viscosity, which compromises jet stability and limits stretching during electrospinning. In contrast, at 15% TEO, a marked reduction in bead formation and an increase in fiber diameter were observed, associated with higher solution viscosity. Furthermore, increasing the tip-to-collector distance in formulations containing 5% and 10% TEO resulted in reduced fiber diameters. At constant voltage of 20 kV, increasing the distance from 15 to 20 cm produced thicker but more uniform fibers. Overall, SEM analysis indicated that the optimal conditions for thermoplastic polyurethane nanofiber production were achieved with 15% TEO, a tip-to-collector distance of 20 cm, and an applied voltage of 20 kV. These conditions yield a uniform morphology and satisfactory nanofiber formation. In another study, the incorporation of OEO at 2% and 3% reduced the viscosity of sodium alginate/PEO polymer solutions compared to OEO-free systems [51]. This effect was attributed to the hydrophobic nature of the oil, which disrupts intermolecular interactions between polymer chains. Neat fibers exhibited larger bead formation, associated with higher viscosity. However, the addition of OEO led to a significant reduction in bead formation, yielding more continuous and uniform fibers [51]. Emulsion systems containing OEO also showed a favorable response to increasing applied voltage, resulting in improved fiber uniformity. Nevertheless, excessive voltage increased fiber diameter and altered morphology. At 22 kV, fibers appeared more separated with small beads; at 26 kV, denser layers of fiber with spindle-like beads were observed; and at 30 kV, thicker fibers with reduced bead size were obtained [51]. Similar trends were observed in electrospun fibers processed at an applied voltage of 17.5 kV. Neat sodium acetate fibers exhibited average diameters of 444–471 nm, whereas the incorporation of LGEO resulted in an increase in fiber diameter, ranging from 493 to 515 nm. Furthermore, increasing the applied voltage to 25 kV led to the formation of beaded fibers with diameters of approximately 600 nm, indicating a deterioration in fiber morphology [27].

Taken together, these findings reinforce the fact that the incorporation of EOs and variations in electrospinning parameters, particularly voltage, significantly influence fiber morphology and diameter. While moderate processing conditions can promote uniform and defect-free fibers, excessive voltage may induce jet instability, leading to bead formation and compromised structural integrity. These results highlight the importance of carefully optimizing both formulation and processing parameters to achieve nanofibers with controlled morphology and desirable functional properties.

## 6. Encapsulation Efficiency (Ee) and Release Kinetics of EO Electrospun Fibers

The assessment of encapsulation efficiency (EE) is a key performance parameter for electrospun polymer matrices. EE corresponds to the fraction of active compounds retained within the fibers relative to the total amount incorporated during the manufacturing process. This is a direct indicator of the system’s loading capacity and its release behavior [116]. EE is influenced by multiple factors, such as the method of EO incorporation, fiber morphology (diameter and porosity), environmental conditions, and physicochemical compatibility between polymer and EO. The latter is one of the most decisive factors in the performance of the encapsulation system [23,38,153]. Low physicochemical affinity can promote the migration of the active compounds toward the fiber surface, causing a rapid initial release (burst release). Conversely, systems with enhanced polymer-active compound compatibility promote a more homogeneous distribution of the active compound within the matrix, favoring sustained and controlled release profiles [23,37,173]. Moreover, several factors play a critical role in determining the release of active compounds from electrospun fibers. These include polymer properties (such as molecular weight and hydrophobicity), fiber characteristics (including diameter and porosity), the nature of the encapsulated agent (e.g., molecular size, solubility, and affinity for the polymer matrix), as well as its distribution within the fiber (surface-located or encapsulated) [174].

The most relevant mechanisms governing the release of active compounds include diffusion, polymer matrix degradation, erosion, and swelling. These could act independently or synergistically depending on the physicochemical properties of the system [175]. In polymers with high structural stability in release medium, diffusion is the predominant mechanism, where the active agent is released in response to concentration gradients from the matrix into the surrounding environment. This process is commonly described by the Higuchi model, which assumes one-dimensional diffusion from a homogeneous matrix and establishes a proportional relationship between the amount released and the square root of time [176]. Table 3 shows the kinetic models that are commonly used in the analysis of drug release behavior. Where *Q_t_* is the cumulative amount of drug released at time *t*, *Q*_0_ is the initial amount of drug released, *Q_t_*/*Q_∞_* is the fraction of drug released at time, *n* is the diffusional exponent, *k_H_*_,_ *k*_0_, *k*_1_, and *k_KP_* are Higuchi dissolution, zero-order, first-order and release rate constant, respectively.

Two main types of diffusion can be distinguished: reservoir-based and matrix-based. In reservoir-based diffusion, the active agent is confined within a core surrounded by a polymeric membrane that regulates its release. In this case, the release kinetics are governed by the permeability and thickness of the outer layer, enabling near-zero-order release profiles (see Table 3) [174,175,176,177,178,179]. In contrast, in matrix-based diffusion, the compound is homogeneously distributed within the polymer network, and its release occurs from the interior to the surface driven by concentration gradients. This mechanism typically results in time-dependent release profiles, often described by the Higuchi model, and may exhibit an initial burst release due to molecules located near the fiber surface [174,176,177,180].

The degradation of the polymeric matrix is particularly relevant in systems based on biodegradable natural polymers. This process involves the cleavage of structural bonds through physical (abrasion, fracture, disintegration), chemical (dissolution, ionization, protonation), or biological mechanisms (pH variations, enzymatic activity, immune response), leading simultaneously to matrix breakdown and active compound release. In such systems, the release kinetics is governed by the degradation rate of the polymer and its interaction with the surrounding environment [176,177]. A related mechanism is erosion, which involves the progressive loss of material mass induced by external factors such as aqueous media, temperature, or radiation. Depending on the dominant process, erosion can be classified as: surface erosion, which promotes near-zero-order release; or bulk erosion, where degradation occurs uniformly throughout the material [176,177,178,181]. Finally, swelling is a key mechanism in hydrophilic biopolymers such as chitosan, gelatin, and alginate. Water uptake induces expansion of the polymer network, increasing the mobility of the encapsulated agent and facilitating its release. This phenomenon is often coupled with diffusion processes and can significantly influence release kinetics [176,177,178].

In complex polymeric systems such as electrospun fibers, multiple release mechanisms may coexist. Therefore, the application of appropriate mathematical models (see Table 3) is essential for the rational design of nanofiber-based systems, particularly in biomedical applications where precise dosing and therapeutic efficacy are required [182,183].

**Table 3:** Kinetic models are commonly used in the analysis of drug release behavior [182,183].

Model	Equation	Description
Higuchi	$Q_{t}=k_{H}\cdot \sqrt{t}$	Describes drug release governed by simple diffusion from insoluble matrices with uniformly distributed drug as a function of time.
Zero order	$Q_{t}=Q_{0}+k_{0}\cdot t$	Exhibits a constant release rate, independent of the remaining drug concentration. It reduces dosing frequency and improves receptor binding. It is often observed in coated matrices.
First order	$\ln Q_{t}=\ln Q_{0}-k_{1}\cdot t$	The release rate is proportional to the amount of drug remaining in the fiber. It is commonly observed when release is rapid or when the matrix undergoes dissolution
Korsmeyer-Peppas	$\frac{Q_{t}}{Q_{\infty }}=k_{KP}\cdot t^{n}$	Shows a power-law relationship between release and time. It is used for systems with mixed mechanisms, including diffusion, polymer relaxation, or erosion.

CS-PEO-CLEO fibers were obtained with 87.6 ± 13.1%, 8.9 ± 0.98% and 79 ± 9.35% of EE, loading and yield, respectively. The CLEO loading is attributed to its hydrophobic nature and low evaporation during the electrospinning process. The percentage of CLEO loaded onto the CS-PEO fibers depends on several factors, such as the nature of the active pharmaceutical ingredient (API) and polymer (hydrophilic/hydrophobic), compatibility, ionic interactions, and method used for drug loading. CLEO release was measured at physiological (7.4) pH, showing rapid release during the initial 12 h due to the hydrophilic behavior of PEO, followed by slow and continuous release over the next 36 h, sustained by the presence of CS [34]. The observed biphasic release profile, characterized by an initial rapid release followed by a controlled phase, suggests that the system fits the Korsmeyer–Peppas model. Indicating a release mechanism dominated by anomalous transport, in which both Fickian diffusion and polymer matrix relaxation coexist. The higher release under acidic conditions further supports the contribution of CS swelling and dissolution in controlling the process [116,173].

Similar values for EE percentages and EO release have been reported in TEO-loaded zein fibers after 180 h, (EE of 75.2% and release of 65%), attributed to the formation of a core–shell structure upon electrospinning onto the zein fiber. Thereby effectively controls the release of TEO compared to its free state, which exhibited a rapid release of 60% after 80 h [35]. The release profile of free TEO zein fibers followed a first-order kinetic behavior, typical of volatile compounds released by evaporation. In contrast, the encapsulated TEO exhibited a sustained release pattern, which was well described by the Higuchi and Korsmeyer–Peppas models, indicating a diffusion-controlled mechanism through the zein fiber matrix. The formation of a core–shell structure during electrospinning effectively acted as a diffusion barrier, which resulted in a significant slowing of volatilization of TEO [184].

Shahbazi et al., 2021 [28], investigated the behavior of carboxymethyl cellulose (0.2%)—gelatin (1%) (CMC-GE) fibers loaded with 0.5%, 1%, and 2% MEO; reporting an EE of 97.83 ± 0.45%, 97.14 ± 0.19%, and 97.33 ± 0.77%, respectively. For the MEO release studies in CMC-GE fibers, the samples were analyzed at 4, 25, and 37 °C for 84 h with measurements taken every 12 h. The results indicate an increase in MEO release proportional to the applied temperature. Furthermore, MEO was rapidly released after 12 h, suggesting that MEO is located near the surface or poorly entrapped within the fiber; however, after 48 h, there is a gradual cumulative release. The release profile of MEO from CMC-GE nanofibrous films, characterized by an initial burst release followed by a sustained release phase and a plateau, is best described by the Korsmeyer–Peppas model. This suggests a non-Fickian transport mechanism governed by the combined effects of diffusion and polymer chain relaxation, which is further influenced by temperature-dependent increases in molecular mobility [116,173].

Fiber morphology is a critical factor in determining release behavior. Homogeneous distribution of EO promotes the formation of smooth and bead-free fibers. Additionally, an increase in fiber diameter due to higher EO content significantly impacts polymer chain entanglement and intermolecular interactions [28,34]. Consequently, a uniform and thicker structure facilitates the initial rapid release, and a denser polymeric network contributes to gradual and sustained release, promoting high encapsulation efficiency. Therefore, morphology, fiber diameter, and polymer composition are key parameters that determine the kinetics of EO release.

## 7. Antimicrobial Activity of Electrospun Fibers Containing EOs Against MDR Pathogens

Research has demonstrated that incorporating EOs into electrospun fibers can remarkably boost their antimicrobial efficacy against multidrug-resistant pathogens. As demonstrated in the literature, significant inhibition against strains such as methicillin-resistant *S. aureus* (MRSA), *P. aeruginosa*, and *ESBL-producing E. coli*, among others, has been reported [28]. As outlined in Table 2, recent studies have examined the incorporation of EOs, including TEO, OEO, CLEO, CEO, TTEO, LEO, MEO and EEO into electrospun matrices. These studies have demonstrated the inhibitory effect of these EOs on various MDR microorganisms.

**Figure 6:**
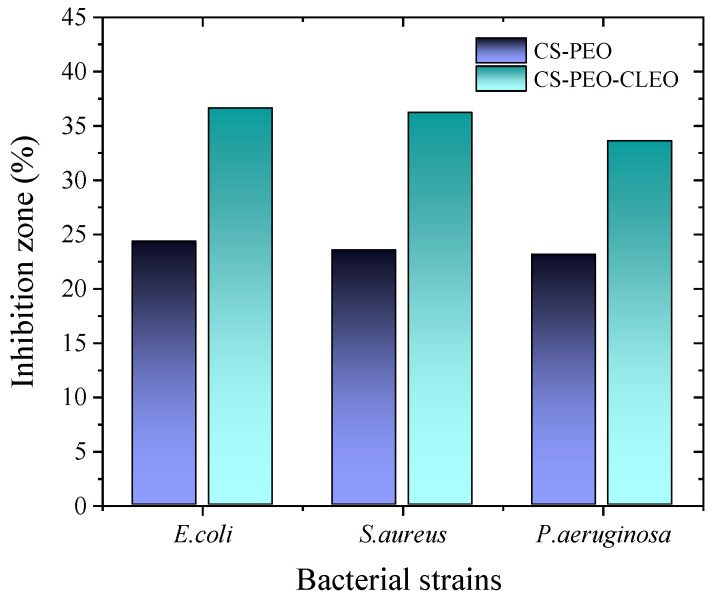
Percentage of inhibition zone of CS/PEO and CS/PEO/CLEO fibers against *E. coli*, *S. aureus*, and *P. aeruginosa* [34].

A critical step in the application of electrospun polymer fibers loaded with EOs is to evaluate their antimicrobial efficacy against multidrug-resistant pathogens. This action will determine their potential for future applications. Hameed et al. (2021) [34] used the Muller-Hinton agar diffusion method to measure antibacterial activity against strains of *S. aureus* (ATCC 29213), *E. coli* (ATCC 8739), and *P. aeruginosa* (ATCC 9027). A methyl thiazolyl tetrazolium (MTT) assay featuring fibroblast cell lines was used to evaluate the cellular viability of electrospun fibers with CS-PEO (5%) and CLEO (0.5% and 1%) incubated at 37 °C for 24 and 48 h, respectively. The results obtained after incubation showed inhibition zones for the CS-PEO fibers of 24.3 ± 3.40, 23.55 ± 5.27, and 23.1 ± 4.6%, and for the CS-PEO-CLEO fibers of 36.6 ± 2.4, 36.2 ± 3.5, and 33.6 ± 5.4% against *E. coli*, *S. aureus*, and *P. aeruginosa*, respectively (see Figure 6).

Regarding cell viability, the assay showed no significant differences in the cytotoxicity of CS-PEO, CS-PEO-CLEO, and the control fibers (*p* > 0.05), suggesting that the concentrations of CLEO combined with the polymers used are safe. This study was further supported by an in vivo wound healing study on 1 × 1 cm circular wounds in 6- to 7-month-old Sprague-Dawley rats weighing 250 ± 20 g. The study examined four groups: Group A (untreated), Group B (CS/PEO fiber), Group C (CS/PEO/CLEO fiber), and finally Group D (commercial formulation). The average wound contraction was measured after 1, 5, and 10 days. The results on days 5 and 10 showed that wound healing was slower in Group A. In contrast, Groups C and D exhibited significantly higher diameters (*p* < 0.001) compared to the untreated group. Group B exhibited lower healing percentage in comparison to Groups C and D, yet it outperformed Group A. Consequently, it has been substantiated that the tested fibers possess healing, antibacterial, and antioxidant properties, attributable to the presence of CS and CLEO [34].

**Figure 7:**
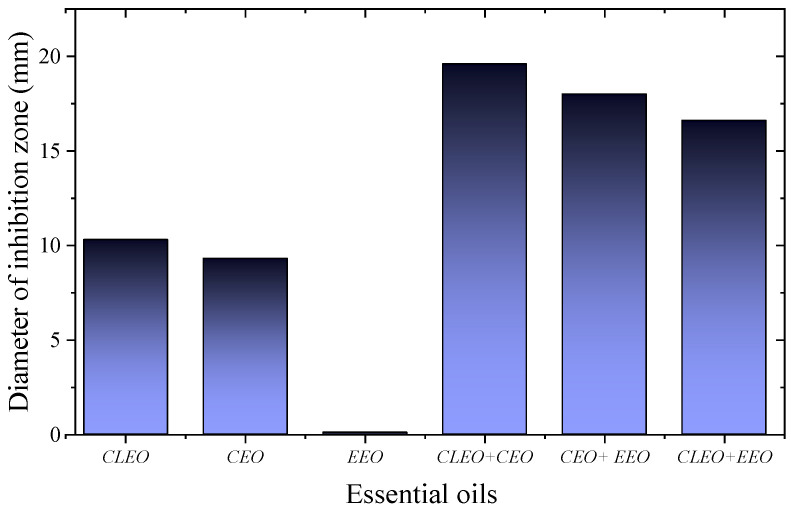
Diameter of inhibition zone of fibers loaded with individual and combined EOs against *S. aureus* [30].

In another study, collagen fibers loaded with CLEO demonstrated 90.83% reduction in colony forming units (CFU) of *E. coli* and a 98.61% reduction in CFU of *S. aureus*. Similarly, fibers loaded with CEO exhibited 100% inhibition of CFU in both strains. This microbiological analysis demonstrates the ability of both EOs to damage the cell membrane, leading to the death of these microorganisms. These properties are attributed to eugenol and cinnamic aldehyde, the main components of the EOs used [172]. In another test, electrospun cellulose acetate fibers doped with CLEO, CEO, and EEO were tested, yielding inhibition zones of 10.3, 9.3, and 0.1 mm, respectively, against *S. aureus* (see Figure 7). Meanwhile, the combination of EOs in the fibers demonstrated superior performance in terms of the inhibition zone, with values of 19.6, 18, and 16.6 mm for the combined samples of CLEO + CEO, CEO + EEO, and CLEO + EEO, respectively. *S. aureus* exhibited heightened sensitivity to the combined CLEO and CEO (see Figure 7). In contrast, for antibiofilm activity, the combined CEO and CLEO + EEO fibers exhibited lower values compared to the fibers loaded with isolated EOs; only the CLEO-CEO system exhibited strong antibiofilm activity [30].

In addition, direct counting of bacterial cells under the action of electrospun cellulose acetate fibers with individual and combined EOs showed faster kinetics for the fiber with CLEO, with gradual increase over time, reducing the cells to 5.8 × 10^−1^ CFU/mL after 60 min, to 3.3 × 10^−1^ CFU/mL after 80 min, and 4.3 × 10^−1^ CFU/mL after 180 min; in contrast, for CEO and EEO, a more subtle response was observed, requiring 180 min to become active against *S. aureus*. In contrast, a rapid reduction in CFU was observed after 90 min of reaction for all combinations of EOs in the following order: the results of the study clearly indicate that CEO + CLEO > CLEO + EEO > CEO + EEO. This finding confirms the superior performance of the CLEO + CEO samples across all evaluated ranges. The study demonstrates synergistic interaction and confirms that eugenol, the common component in both EOs, is an important antibacterial agent [30]. In another study, two common strains involved in bacterial wound infections, MRSA and *K. pneumoniae*, were subjected to contact tests with electrospun alginate fibers containing 2% and 3% OEO, cross-linked with 4% (*w*/*w*) CaCl_2_ for 10 min, demonstrating that inhibition zones averaging between 6 and 8.5 mm were produced in the fibers containing 2% and 3% OEO against MRSA; however, the fibers with additional OEO showed better results against both strains, indicating that electrospun fiber technology represents a successful delivery system for OEO, which could be used in wound care management (dressings) [51].

In a study with electrospun zein fibers doped with TEO at 4%, inhibition zones of 15.54 ± 0.24 mm for *B. cereus*, 8.12 ± 0.52 mm for *E. coli*, and no visible growth for *A. fumigatus* were observed. These results suggest that fungi were the most susceptible to the treatment, followed by Gram-positive bacteria, while Gram-negative bacteria showed lower sensitivity. This reduced sensitivity may be attributed to the TEO concentration used or to the presence of an outer membrane in Gram-negative bacteria [35]. Additionally, the uniform morphology of TEO-loaded zein fibers favored the formation of a core–shell structure during electrospinning, enabling efficient encapsulation and sustained release of the essential oil. This controlled release profile maintained effective TEO concentrations over extended periods, which was reflected in significant antimicrobial activity, particularly against *A. fumigatus* and Gram-positive bacteria. In contrast, the rapid release of TEO in its free form limits its long-term efficacy, highlighting that fiber architecture and release kinetics are key determinants in enhancing antimicrobial performance [35].

Similarly, the antimicrobial activity of electrospun fibers made from the Ecovio^®^ (EC) polymer blend, composed of PLA and poly(butylene adipate-co-terephthalate) (PBAT), incorporated with TTEO at concentrations of 0.5, 1, and 1.5%, was evaluated. After 24 h of contact with *S. aureus* and *P. aeruginosa* strains, it was observed that the surfaces of the fibers with EC/TTEO0.5, EC/TTEO1.0, and EC/TTEO1.5% were not completely covered by *P. aeruginosa*, in contrast with the control EC and PS fibers, where total coverage was evident. These results suggest that the incorporation of TTEO gives the fibers a potential anti-adherent effect against *P. aeruginosa*. However, it is necessary to further evaluate parameters such as the minimum effective concentration and dose–response relationship to confirm and quantify this effect [33]. In the disk diffusion assay of EC/PS/TTEO fibers, no inhibition zones were found against any strain, suggesting that the presence of irregularities and heterogeneity in the fibers interferes with the development of this study. The authors indicate that TTEO could interact with the fibers through Van der Waals forces, generating dipole–dipole interactions or London dispersion, which inhibit the release of TTEO into the diffusion medium, preventing the formation of inhibition halos [33].

Several studies have observed antimicrobial activity against strains of *E. coli*, *S. aureus*, *L. monocytogenes*, *B. subtilis*, *B. cereus* and *S. typhimurium* in electrospun fibers containing MEO, suggesting that this activity is primarily related to the interaction of its phenolic compounds with the cell surface, binding to nucleic acids and disrupting DNA expression [96,107]. A study using fibers loaded with 0.5, 1, and 2% MEO and containing 0.2% carboxymethylcellulose (CMC) and 1% GE confirmed antimicrobial activity for the pure CMC polymer and the CMC/GE/MEO mixture. Pure CMC was more effective than the mixture, possibly due to interactions between the components. A study of CMC/MEO is required to compare the effectiveness of the EOs [148]. Conversely, a study involving 4.8% polycaprolactone (PCL)/GE with 1.5%, 3% and 6% CLEO demonstrated effectiveness against *E. coli* and *S. aureus* for up to 48 h of incubation, with a decrease in bacterial viability observed compared to PCL fiber alone. Furthermore, the authors suggest that the activity increases with a higher MEO content in the PCL fiber, with the 6% PCL/CLEO composition exhibiting the lowest bacterial viability [29].

A study analyzed the effectiveness of PLA fibers treated with limonene-based antimicrobial agents against *E. coli*, *S. aureus* and *B. subtilis*. After 24 h of incubation, no colonies were observed on plates containing limonene fibers, whereas colonies were present on those containing PLA. The storage time of the fibers did not affect the antibacterial activity of the samples; the PLA–limonene fibers remained effective for four weeks, just like freshly prepared fibers. The effectiveness of limonene against pathogens is due to its chemical properties, which alter cell wall integrity and permeability, inhibit respiration and regulate bacterial enzymes and metabolites. These effects have been observed in a wide range of microorganisms, including multidrug-resistant ones, making limonene a promising natural alternative to antibiotics [31].

Considering the studies described above, it is important to review the status of EOs in biomedical applications. Despite the growing interest in EOs as natural antimicrobial agents, most available studies have focused on evaluating their activity in their free form. Here, a broad spectrum of activity against MDR bacteria has been demonstrated [116,119,126,128,130,131,132,133]. However, direct application is limited by their high volatility, chemical instability, low aqueous solubility, and susceptibility to oxidation, which compromise sustained efficacy and therapeutic reproducibility [185,186]. In this regard, nanoencapsulating EOs in electrospun polymeric fibers is a promising technological strategy for improving their physicochemical stability, protecting their bioactive compounds and modulating their release. Nevertheless, the number of studies integrating EOs into electrospun nanofibers is considerably lower than that for non-encapsulated EOs, indicating a significant opportunity to develop more robust and clinically viable systems [187,188,189].

Furthermore, existing research on electrospinning systems has predominantly focused on a limited set of model strains, primarily *E. coli*, *S. aureus* and *P. aeruginosa*. This has left their potential against other clinically relevant pathogens insufficiently explored [27,28,33,34,35,36,51,97,172]. This includes strains that produce extended-spectrum β-lactamases (ESBLs) and carbapenemases, as well as microorganisms that have a high capacity for biofilm formation. This limited microbiological diversity hinders our ability to comprehensively understand the performance of these materials in complex clinical settings, where AMR and biofilm formation play a decisive role in treatment failure [108].

Therefore, there is a significant gap in the literature regarding the systematic evaluation of EO-loaded electrospun fibers against a broad spectrum of MDR microorganisms, as well as in establishing correlations between composition, morphology, encapsulation efficiency, and antimicrobial efficacy. Addressing this gap would not only optimize the design of functional materials but also strengthen their potential application in medical textiles, antimicrobial coatings, and preventive strategies against the growing crisis of AMR [39].

## 8. Limitations and Challenges for Biomedical Applications

Despite growing interest in the use of electrospun fibers functionalized with bioactive molecules in the medical field, where there is rising threat from MDR pathogens, including *S. aureus*, *P. aeruginosa*, and *E. coli*, this technology is still considered emerging, and its clinical adoption faces significant obstacles. The main challenges include scientific, technical and regulatory limitations that hinder progress towards real-world applications [22,75,190].

One critical aspect is the biocompatibility of the system, as both the polymeric material and bioactive compounds must demonstrate cellular safety and absence of cytotoxic effects [47,191]. Furthermore, high loads of EOs or other active agents can compromise the physicochemical stability of the electrospun fibers and lead to variability in release profiles [23,37]. This is compounded by the reproducibility of the process, which depends on sensitive parameters such as solution viscosity, applied voltage, needle-collector distance, and ambient humidity; all these factors affect the diameter, surface morphology, and homogeneous distribution of the active compound within the polymeric fiber, thereby limiting its standardization in biomedical environments.

At the experimental level, the lack of standardization in preclinical evaluation protocols, including toxicological studies, cytocompatibility, antimicrobial efficacy, system stability and in vivo behavior, remains a challenge [22,72,192]. Furthermore, the transition from laboratory to commercial applications requires the overcoming of additional challenges, including optimizing polymer–EO interactions, developing formulations with long-term chemical stability, and scaling up the electrospinning process to ensure reproducibility and quality control, while also complying with strict regulatory frameworks to ensure the safety, hygiene, and traceability of the final product [193,194]. It is recognized that electrospinning faces limitations in scalability and process control, which constitute one of the main obstacles to its implementation in the production of medical devices and controlled-release systems [194,195].

These limitations highlight the need for optimization and validation strategies, incorporating emerging technologies such as green electrospinning (using non-toxic solvents), integration with sensors and smart systems, automated control via machine learning and advanced characterization methods. Overcoming these challenges is essential to ensuring the safety, efficacy and scalability of electrospun materials intended for medical use, as well as consolidating their transition towards clinical and commercial applications [195]. These obstacles highlight the importance of developing optimization and validation strategies that strengthen their applicability in clinical settings. These considerations are fundamental to ensuring the safety, efficacy and scalability of materials intended for medical use [40,196].

## 9. Future Outlook

Advances in computational tools, molecular modelling and artificial intelligence are radically transforming the design of functional electrospinning systems. These emerging technologies will optimize interactions between polymeric matrices and EOs, enable more accurate prediction of release profiles and facilitate the design of multifunctional platforms with highly integrated antimicrobial, anti-inflammatory and regenerative properties [197,198]. Similarly, the application of silico methods will facilitate the systematic exploration of understudied plant species, accelerating the identification of new bioactive compounds and their incorporation into advanced delivery systems [23,191].

In the long term, strategically implementing electrospun fibers loaded with EOs could significantly help to mitigate global AMR, in line with international initiatives that aim to reduce reliance on conventional antibiotics. However, achieving this will require an interdisciplinary approach integrating materials engineering, polymer chemistry, microbiology, pharmacology and clinical sciences, as well as clear regulatory frameworks ensuring safety, standardization, traceability and industrial-scale production [198,199].

The intrinsic properties of nanofibers produced by electrospinning, such as, high surface-to-volume ratio, adjustable porosity, compositional versatility and functionalization potential, open up opportunities in diverse sectors, including advanced filtration, protective clothing, tissue engineering, wound dressings, controlled-release systems, chemical and optical sensors, and smart materials for environmental applications [173,197]. Notwithstanding, transitioning them to the clinical setting requires ensuring the biocompatibility of the entire system, preserving the biological activity of the encapsulated compounds and optimizing encapsulation efficiency to guarantee consistent and safe performance [23,25].

The antibiofilm properties of these systems are particularly valuable for developing functional coatings for medical devices such as catheters, probes, prostheses and implants, as microbial colonization is a common cause of treatment failure in these devices [198,199]. In hospital settings, these fibers could be incorporated into medical textiles, such as surgical gowns and face masks, as well as contact surfaces, thereby extending the duration of the antiseptic effect and reducing the transmission of pathogens [37,191]. Outside the biomedical field, there are promising applications emerging in active food packaging, air and water filtration, and agricultural materials, which further highlight their importance in terms of public health and sustainability [23,190]. Overall, electrospun fibers loaded with EOs represent a technological platform with enormous translational potential. Their future development will depend on the strategic integration of digital tools, the optimization of scalable processes, and regulatory harmonization that will enable these innovations to be transformed into practical, sustainable solutions with high social impact [26,200].

## 10. Conclusions

AMR continues to be consolidated as one of the most critical threats to global public health, particularly in the face of MDR pathogens from the ESKAPE group, recognized for their high capacity for therapeutic evasion and persistence in hospital environments. In this context, EOs have emerged as broad-spectrum antimicrobial agents due to their multifactorial mechanisms of action, including membrane disruption, metabolic interference, and modulation of bacterial signaling systems. Nevertheless, a systematic correlation between these biological properties and advanced encapsulation strategies remains insufficiently established. Electrospinning offers significant advantages, including a high surface-to-volume ratio, morphological tunability, protection against volatilization and degradation, and the potential for controlled release. Key variables such as polymer composition, EO incorporation strategy, polymer–EO physicochemical compatibility, and fiber morphology play a decisive role in determining encapsulation efficiency and antimicrobial performance.

Despite these recognized advantages and promising antimicrobial mechanisms of EOs, translating their potential into robust, reproducible, and clinically applicable materials through electrospinning remains fraught with unresolved challenges. These include limited exploration of EO diversity and natural polymeric matrices along with challenges such as weak intermolecular interactions, polarity mismatch, phase separation and variability in electrospinning conditions, all of which undermine structural homogeneity, reproducibility, and antimicrobial efficacy. Another major gap is the lack of biological validation using clinically relevant MDR strains, since there are few preclinical or clinical studies addressing safety and therapeutic performance. To advance real-world biomedical applications, future research must adopt systematic approaches, broader EO and polymer selection, and clinically relevant models.

Overall, electrospun natural polymeric fibers loaded with EOs represent a multifunctional and highly promising platform for biomedical applications, including wound healing, medical textiles, and antimicrobial coatings. The integration of interdisciplinary strategies, such as computational modeling, material informatics, and artificial intelligence could enable the rational design of optimized systems, thus accelerating their development and strengthening their role as sustainable and effective alternatives to combat the global AMR crisis.

## Figures and Tables

**Table 1:** Aspects and variables of the electrospinning process and its effects on the electrospun fibers [31,32,56].

Aspect	Variables	Increase/Decrease in the Variable	Effect on the Electrospun Fibers
Internal or solution-related	Concentration	↑ ↓	Reduction in diameter Does not form fibers, pearl formation
	Surface tension	↑ ↓	Appearance of defects in fibers Reduces pearl formation, smooth fibers
	Conductivity	↑ ↓	Thin fibers Thicker fibers with lack of uniformity
	Dielectric effect	↑	Reduces pearl formation, reduction in diameter
Process	Voltage	↑ ↓	Thinner fibers without pearls Low momentum of solution arrival at the collector
	Outlet flow	↑ ↓	Fiber diameter increases Generates Taylor cone, defect-free fiber
	Distance Injector-Collector	↑ ↓	Fibers break or are not collected Formation of droplets or wet fibers
Environmental	Humidity	↑ ↓	Generates porous fibers 10% to 40% optimal for fiber formation
	Temperature	↑	May increase evaporation and reduce viscosity

An increase or decrease in the variable is represented by "↑" or "↓", respectively.

**Table 2:** Effect of polymer and EO composition on the physicochemical and antimicrobial properties of electrospun natural polymeric fibers loaded with EOs.

Polymer/ Concentration	EO/ Concentration	Active Compounds	Electrospinning Solution Parameters	Morphology	Diameter	Microorganism	Best Antimicrobial Result	Antimicrobial Mechanism	Ref.
Carboxymethylcellulose (CMC)/2% and gelatin (GE)/10%	MEO (*Mentha* spp.)/2%	β-pinene, β-pinene and limonene, menthone or 5-methyl-2-(1-methylethyl)cyclohexanone, menthol	124.99 cP; 396.54 µS cm^−1^; 48.92 mN m^−1^	Thin, uniform, with microspheres	454.76 ± 0.69 nm	*A. baumannii*, *P. aeruginosa*, *S. aureus*, *L. monocytogenes*, *C. albicans* and *A. niger*	Diameter of inhibition zone: 7.43 mm against *S. aureus.*	Inhibition of toxin production, elastase, proteases, pyocyanin, and chitinase.	[28]
Chitosan with polyethylene oxide/5%	CLEO (*Syzygium aromaticum*)/1%	Eugenol, β-caryophyllene, α-humulene	1871 cP; 2228 µS cm^−1^	Soft, smooth, with some beads	189 ± 43 nm	*S. aureus*, *E. coli.* and *P. aeruginosa*	Inhibition zone: 36.6% against *E. coli.*	Anti-QS and anti-biofilm. QS-Inhibits activator motility.	[34]
Zein/30%	TEO (*Thymus vulgaris*)/4%	Thymol, carvacrol, linalool, α-terpineol, 1,8-cineole, p-cymene.	-	Uniform and smooth	402.3 ± 26.6 nm	*B. cereus*, *E. coli.* and *A. fumigatus*	Diameter of inhibition zone: 15.54 mm against *B. cereus*	Inhibition of enterotoxins, changes in fatty acid composition, anti-QS and biofilm.	[35]
Polycaprolactone/12%	TEO *(Thymus vulgaris*)/5%	Thymol	431.7 cP	Uniform and smooth	131.0 ± 7.9 nm	*S. aureus*, *S.epidermidis*, *E. coli*	Bacterial reduction approaching 100% against *S. aureus*, *S. epidermidis*, *E. coli*, and *P. aeruginosa*	Damage to cell membrane integrity, reduced biofilm formation, and DNA destabilization	[36]
Polyvinyl alcohol/10% *w*/*v* and gellan/1% *w*/*v*	Cinnamaldehyde	Cinnamaldehyde	572.37 cP; 67.17 µS cm^−1^	Thin and porous	278.5 ± 57.8 nm	*Candida albicans*, *Candida glabrata*, *S. aureus* and *P. aeruginosa*	Reduced 72% of *S. aureus* and 43% of *P. aeruginosa.*	Disruption of the cytoplasmic membrane	[106]
Polyurethane/10%	TEO/15%	Carvacrol, thymol, linalool, cineole	524.8 cP; 34.95 mN m^−1^	Thin entangled	~353 nm	-	-	-	[37]
Hydrolyzed collagen/10%	CLEO/10% and CEO/10%	CLEO: Eugenol; CEO: cinnamic aldehyde	1623 cP; 8.5 pH	Thin, uniform	429.6 nm (CLEO) and 463.2 nm (CEO) on waxed paper	*E. coli*, *S. aureus* and *C. albicans*	Reduction in colony forming units: 98.61% (CLOE) and 100% (CEO) against *S. aureus.*	Disruption of the cytoplasmic membrane	[172]
Cellulose acetate (CA)/~8.8% *w*/*v* and poly(ethylene oxide) (PEO) ~0.88% *w*/*v*	CEO, CLEO, and EO of eucalyptus (EEO)/~16.7% *v*/*v*	CLEO: Eugenol; CEO: cinnamic aldehyde, eugenol and linalool; EEO: limonene, eucalyptol and pinene	-	Regular and cylindrical	2.13 ± 0.49 µm (CLEO); 1.98 ± 0.49 µm (CEO).	*S. aureus*	Diameter of inhibition zone: 10.3 mm (CLEO); 9.3 mm (CEO); 19.6 mm (CEO + CLEO) against *S. aureus.*	-	[30]
Polylactic acid (PLA)/14%	(R)-(+)-Limonene/10%	(R)-(+)-Limonene	-	Thin, uniform and smooth	633 ± 304 nm	*E. coli*, *S. aureus* and *B. subtilis*	100% inhibition against *E. coli*, *S. aureus* and *B. subtilis*	-	[31]
Polyethylene oxide	LEO/9%	Citral, Geranial-Neral, Geranial, Geraniol, among others	257 cP (10 s^−1^); 1007 µS cm^−1^	Uniform and bead-free	0.54 ± 0.05 μm	*E. coli*, *S. aureus*, *L. curvatus*, *S. typhimurium* and *Aspergillus niger*	Diameter of inhibition zone: 22.49 mm against *Aspergillus niger*	-	[32]
Cellulose acetate/17%	*Lemon myrtle*/20%	Citral (3,7-dimethyl-2-7-octadienal) geranial, linalool, and β-myrcene	~650 cP (10 s^−1^); 6.23 µS cm^−1^	Uniform diameters	515 nm	*E. coli* and *S. aureus*	100% inhibition against *E. coli* and *S. aureus*	-	[27]
Poly(lactic acid) and poly(butylene adipate-co-terephthalate	TTEO *(Melaleuca alternifolia*)/1.5 mL	Terpinen-4-ol, γ-terpinene, and α-terpinene	6.9 cP; 0.74 µS cm^−1^; 30.1 mN m^−1^	Heterogeneous structures with beads	278 ± 59 nm	*P. aeruginosa*, *S. aureus*	Pure TTEO: 99.99% inhibition of *S. aureus* and *P. aeruginosa* at 7.5 mg/mL	Damage to the bacterial cell membrane.	[33]
Sodium alhinate/3.75% and polythylene oxide/4%	OEO (*Lippia graveolens*)/3%	p-cymene, thymol, carvacrol, γ-terpinene	-	Cylindrical fibers	38–105 nm	*S. aureus*, *MRSA*, *K. pneumoniae*, *S. enterica*	Mean inhibitory zones between 6 and 8.5 nm against MRSA, *K. pneumoniae*, *S. enterica* and *L. monocytogenes*	-	[51]
Polyvinyl alcohol and soy protein isolate/50:50 ration	CEO *(Cinnamon zeylanicum*)/20% *w*/*v*	Cinnamaldehyde, carvacrol	-	Uniform and bead-free	130 nm	*E. coli*, *S. aureus*	Reduction in colonies (CFU): 72% against *S. aureus*	Damage to the bacterial membrane	[97]

## Data Availability

No new data were created or analyzed in this study.
